# Activation of Wnt/β‐catenin signaling by lithium chloride attenuates d‐galactose‐induced neurodegeneration in the auditory cortex of a rat model of aging

**DOI:** 10.1002/2211-5463.12220

**Published:** 2017-04-25

**Authors:** Ming‐Yu Xia, Xue‐Yan Zhao, Qi‐Lin Huang, Hai‐Ying Sun, Chen Sun, Jie Yuan, Chang He, Yu Sun, Xiang Huang, Wen Kong, Wei‐Jia Kong

**Affiliations:** ^1^ Department of OtolaryngologyUnion HospitalTongji Medical CollegeHuazhong University of Science and TechnologyWuhanChina; ^2^ Institute of OtorhinolaryngologyUnion HospitalTongji Medical CollegeHuazhong University of Science and TechnologyWuhanChina; ^3^ Department of EndocrinologyUnion HospitalTongji Medical CollegeHuazhong University of Science and TechnologyWuhanChina

**Keywords:** aging, auditory cortex, Bmi1, presbycusis, Wnt/β‐catenin signaling

## Abstract

Degeneration of the central auditory system, which is characterized by reduced understanding of speech and source localization of sounds, is an important cause of age‐related hearing loss (presbycusis). Accumulating evidence has demonstrated that Wnt/β‐catenin signaling plays an essential role in the development of the auditory system but its potential role in presbycusis remains unclear. In this study, we used a rat model of aging, created by chronic systemic exposure to d‐galactose (d‐gal), and explored changes in Wnt/β‐catenin signaling in the auditory cortex. A decrease in Wnt/β‐catenin signaling in the auditory cortex was found in both naturally aging and d‐gal‐mimetic aging rats, as indicated by increased GSK3β activity and decreased β‐catenin activity. Moreover, lithium chloride (Licl), an activator of Wnt signaling pathway, was administered long term to 15‐month‐old d‐gal‐treated rats. Activation of Wnt/β‐catenin signaling by Licl attenuated d‐gal‐induced auditory cortex apoptosis and neurodegeneration. Bmi1, a transcription factor implicated in antiaging and resistance to apoptosis, can be modulated by β‐catenin activity. Here, we showed that the expression of Bmi1 was reduced and the expression of its downstream genes, *p16* ^*INK*^ ^*4a*^, *p19* ^*Arf*^, and *p53* were increased in the auditory cortex both of naturally aging and d‐gal‐mimetic aging rats. In addition, Licl significantly increased Bmi1 expression and reduced p16^INK^ ^4a^, p19^Arf^, and p53 expression. Our results indicated that decreased Wnt/β‐catenin signaling might participate in the pathogenesis of central presbycusis through modulating the expression of Bmi1. Wnt/β‐catenin signaling might be used as a potential therapeutic target against presbycusis.

**Abbreviations**
AHL: age‐related hearing loss
Bmi1: B lymphoma Mo‐MLV insertion region 1
CD: common deletion
Cyclin D1: cell cycle protein D1
d‐gal: d‐galactose
GSK3β: glycogen synthase kinase 3β
Licl: Lithium chloride
LSCM: laser‐scanning confocal microscope
mtDNA: mitochondrial DNA
PBS: phosphate‐buffered saline
TCF/LEF: T‐cell factor/lymphoid enhancer‐binding factor
TUNEL: terminal deoxynucleotidyl transferase‐mediated deoxyuridine 5′‐triphosphate nick‐end labeling

Aging is a natural phenomenon associated with a progressive degeneration of physiological function and increased vulnerability to disease. Age‐related hearing loss (AHL), also known as presbycusis, is a highly prevalent sensorineural hearing impairment caused by aging. Typically, presbycusis is characterized by reduced ability to hear sound and understand speech, impaired central processing of acoustic information and reduced ability to localize sound sources 1, 2. It is generally acknowledged that presbycusis was caused by the degeneration of peripheral auditory system. The loss of hair cells and dysfunction of the stria vascularis were found to be the main pathological manifestation of peripheral presbycusis 3. Growing evidences demonstrate that the degeneration of the central auditory system with aging also exerts a key role in the process of the pathogenesis of presbycusis 4, 5. The main reason is that age‐related degeneration of the central auditory system (central presbycusis) can affect speech understanding and sound source localization 6. In recent years, there has been tremendous progress in identifying the clinical conditions and pathophysiology of central presbycusis 7, 8, 9. However, the molecular mechanisms involved in central presbycusis remain elusive.

Wnt/β‐catenin signaling is also referred to as canonical Wnt signaling. Extracellular Wnt ligands bind to the Frizzled family receptors (FZDs), leading to the disruption of an intracellular complex that consists of glycogen synthase kinase 3β (GSK3β), Axin, and adenomatous polyposis coli (APC). This prevents phosphorylation of the transcription factor β‐catenin by GSK3β, allowing the stabilized state of β‐catenin to accumulate in the cytoplasma and then migrate to the nucleus. Once located in the nucleus, β‐catenin interacts with T‐cell factor/lymphoid enhancer‐binding factor (TCF/LEF) to drive the expression of Wnt target genes, such as *cyclinD1* and *c‐myc* 10, 11, 12. Lithium chloride (Licl) is an agonist of the canonical Wnt signaling that can inhibit GSK3β activity and thereby stabilize free cytosolic β‐catenin effectively 13. Wnt signaling is known for its role in embryologic development, self‐renewal of stem cells, tumorigenesis, and maintenance of mature tissues 14, 15. It was later found that the downregulation of Wnt signaling contributes to the pathogenesis of certain aging‐associated diseases. Decreased β‐catenin expression contributes to age‐related osteoporosis 16, 17. Dysfunctional Wnt signaling is also implicated in the development of Alzheimer's disease (AD). Increased β‐catenin activity has the ability to provide neuronal protection by reducing β‐amyloid toxicity 18. Furthermore, canonical Wnt signaling has been implicated in the development of the auditory organs and maintenance of the function of auditory system 19, 20, 21. However, the relation between Wnt/β‐catenin signaling and presbycusis is still unknown.

B lymphoma Mo‐MLV insertion region 1 (Bmi1), a member of the Polycomb group family, is involved in resistance to apoptosis and antiaging 22, 23. Interestingly, recent evidence demonstrated that Wnt/β‐catenin signaling regulates Bmi1 expression in colon cancer cells 24. *Bmi1* ^−/−^ mice exhibit progressive postnatal growth retardation, neurological abnormalities, and a reduced lifespan 25, 26. Recent studies have shown decreased Bmi1 expression in the human and mouse central nervous system (CNS) during aging, and its absence causes hypersensitivity to neural apoptosis and premature neurodegeneration 27, 28, 29. Its overexpression can prevent cell senescence by repressing the *Ink4a/Arf* locus, which encodes the p16^INK4a^ and p19^Arf^ proteins 30, 31, 32, 33. p16^Ink4a^ promotes Rb activation, while p19^Arf^ regulates p53 activity. Increased expression of p16^INK4a^ and p19^Arf^ with aging in a variety of tissues, leads to tissue degeneration and aging 34, 35. However, whether Bmi1 is involved in the process of central presbycusis remains obscure. Due to Bmi1 playing a critical role in the aging process of CNS, exploring the regulation of Bmi1 is essential during the development of central presbycusis.

In our previous studies, we established a mimetic rat model of aging using overdoses of d‐galactose (d‐gal). And common deletion (CD), the common mtDNA deletion (4977 bp deletion in human, 4834 bp deletion in rats), have been reported to accumulate gradually in various tissues during aging 36, 37. In the d‐gal mimetic rat, CD was found accumulated both in the peripheral and central auditory system during aging 38, 39. Furthermore, studies indicated CD accumulation is highly related to the development of presbycusis both in human and experimental animals and it will increase the susceptibility of presbycusis 38, 40. Herein, we detected the expression of β‐catenin and GSK3β in the central auditory cortex at different ages in natural aging and d‐gal mimetic aging rats. To further investigate the possible role of Wnt/β‐catenin signaling in neuronal survival in the auditory cortex during aging, we treated the 15‐month‐old d‐gal rats with Licl to activate Wnt/β‐catenin signaling, and detected the CD level, the apoptosis level, and the ultrastructural changes in the auditory cortex. The expression of Bmi1 and its downstream genes, *p16* ^*INK4a*^, *p19* ^*Arf*^, and *p53,* were also detected to explore the possible mechanism of presbycusis in the central auditory system.

## Results

### Age‐related decrease in Wnt/β‐catenin signaling in the auditory cortex

**Figure 1:**
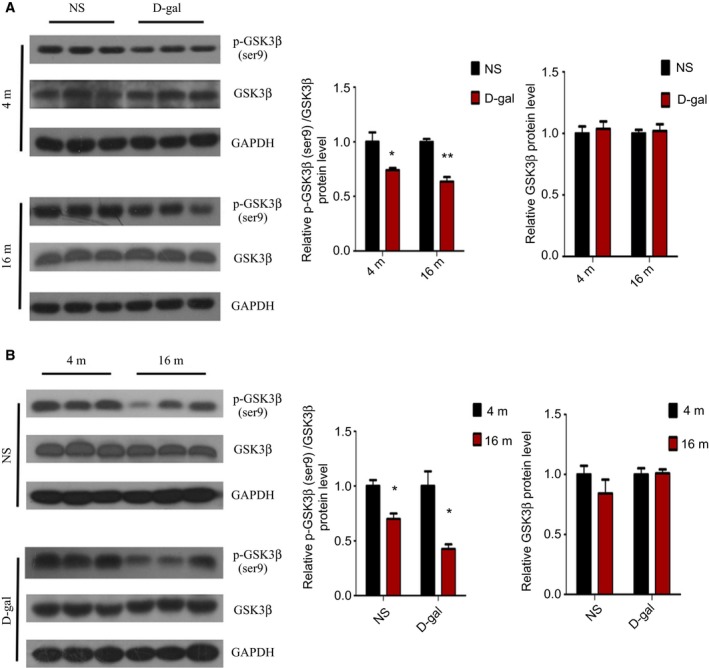
Age‐related decreases in phosphorylation of GSK3β at ser^9^ in the auditory cortex. (A) Western blot analysis of p‐GSK3β (ser^9^) and GSK3β protein expression in d‐gal rats vs NS rats. (B) Western blot analysis of p‐GSK3β (ser^9^) and GSK3β protein expression in 16‐month‐old rats vs 4‐month‐old rats. *P < 0.05, **P < 0.01.

To explore the long‐term changes of Wnt/β‐catenin signaling in the auditory cortex during aging, we examined the activity of GSK3β and β‐catenin between the different groups. It is well known that GSK3β is one of the main negative regulators of canonical Wnt signaling, and p‐GSK3β (ser^9^) is a form of inactivated GSK3β 41. To determine whether aging had an effect on GSK3β activity, western blot analysis was performed. As shown by Fig. 1A, in comparison with the 4‐month‐old NS or d‐gal rats, the level of p‐GSK3β (ser^9^) was decreased in the d‐gal rats at the ages of 4 months (*P* < 0.05) and 16 months (*P* < 0.01). However, the protein levels of GSK3β showed no substantive change. Similarly, the decreased phosphorylation of GSK3β at ser^9^ was also observed in the 16‐month‐old NS group and 16‐month‐old d‐gal rats compared with the 4‐month‐old NS and 4‐month‐old d‐gal rats, respectively (*P* < 0.05), and GSK3β protein expression showed no change (Fig. 1B). These results indicated an age‐related increase in the activity of GSK3β in the auditory cortex.

**Figure 2:**
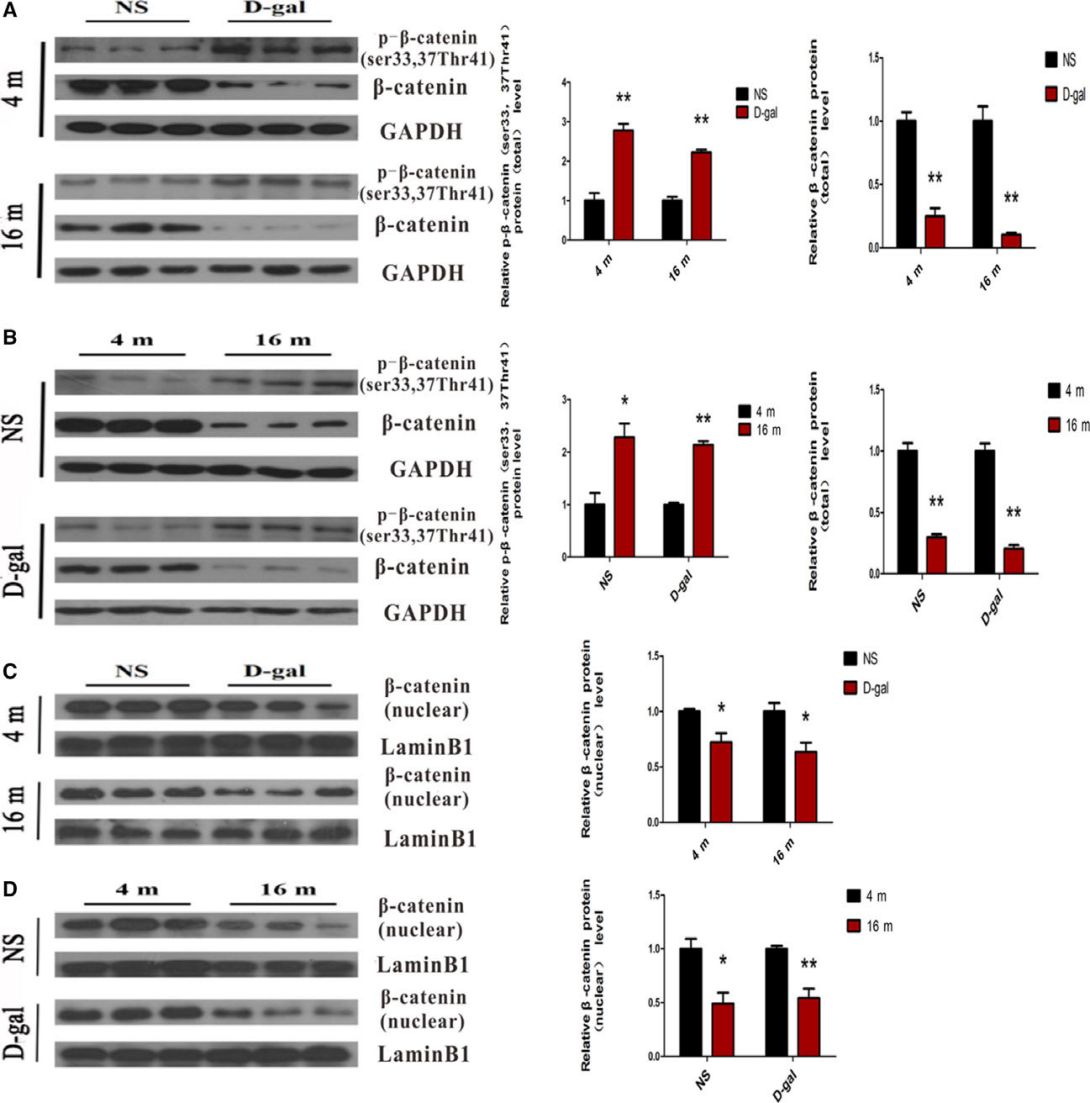
Age‐related improvement in phosphorylation of β‐catenin by GSK3β led to decreased β‐catenin activity in the auditory cortex. (A) Western blot analysis showed the level of p‐β‐catenin (ser^33, 37^Thr^41^) and total β‐catenin protein expression in d‐gal rats vs NS rats. (B) Western blot analysis showed the level of p‐β‐catenin (ser^33, 37^Thr^41^) and total β‐catenin protein expression in 16‐month‐old rats vs 4‐month‐old rats. (C) Western blot analysis showed β‐catenin in nuclear extracts after d‐gal injection. (D) Western blot analysis showed β‐catenin in nuclear extracts in 16‐month‐old rats vs 4‐month‐old rats. *P < 0.05, **P < 0.01.

**Figure 3:**
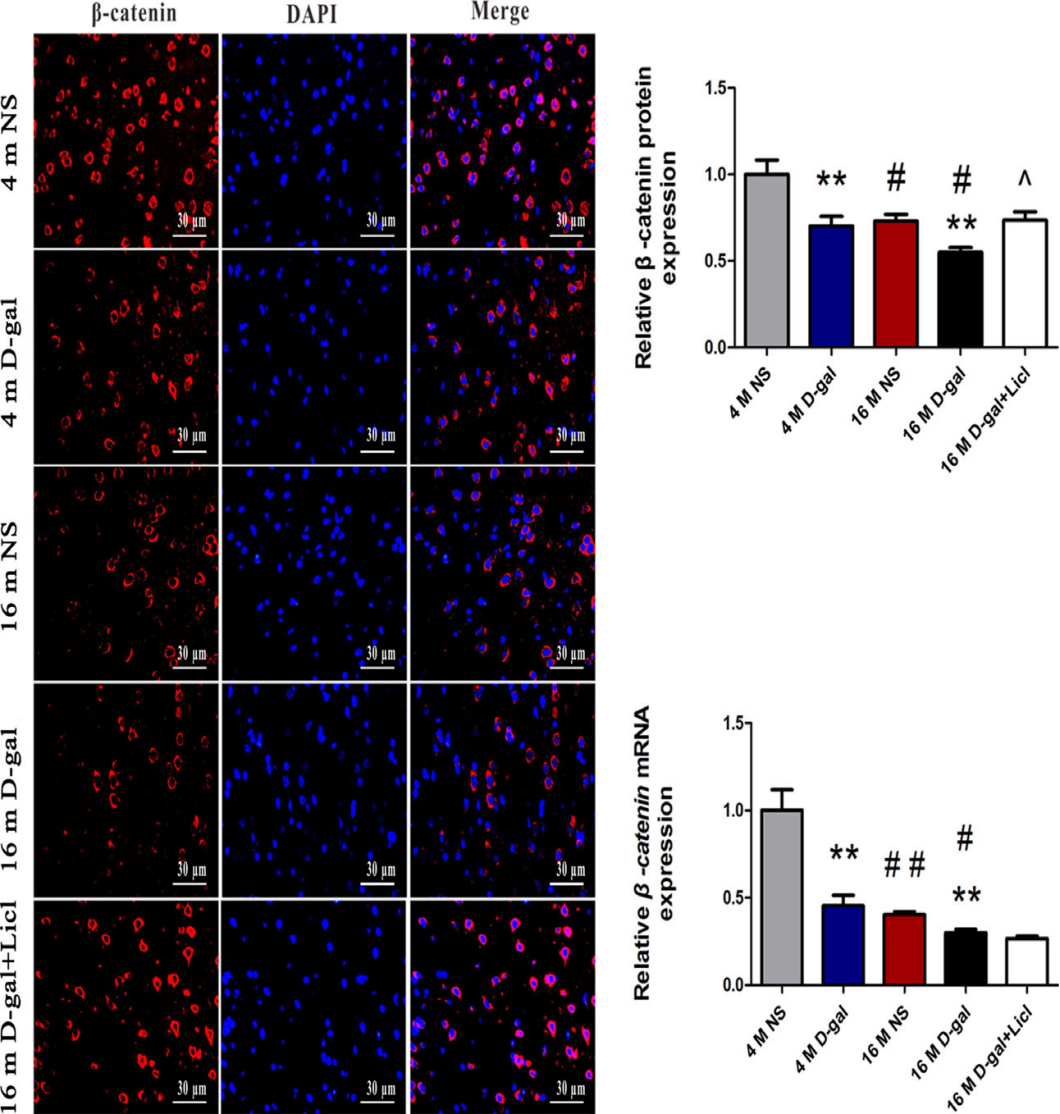
Age‐related decrease in β‐catenin expression and long‐term treatment of Licl improved β‐catenin expression in the auditory cortex. (A) Immunofluorescence analysis of β‐catenin expression in 4‐ and 16‐month‐old NS, d‐gal rats, and 16‐month‐old d‐gal + Licl rats. (B) RT‐PCR showed β‐catenin expression in 4‐ and 16‐month‐old NS, d‐gal rats, and 16‐month‐old d‐gal + Licl rats. The results are expressed as the mean ± SEM (*n* = 5 for each group). *P < 0.05, **P < 0.01 vs NS group. #*P* < 0.05, ##*P* < 0.01 vs 4‐month‐old group. ^*P* < 0.05 vs 16‐month‐old d‐gal group.

In the absence of Wnt stimulation, the amount of cytoplasmic β‐catenin that translocated into the nucleus was decreased, which was due to the phosphorylation of β‐catenin at Ser^33^, Ser^37^, and Thr^41^ by GSK3β 42, 43. To further investigate whether activation of GSK3β could induce altered β‐catenin activity in the auditory cortex, western blot analysis revealed that the protein level of p‐β‐catenin (ser^33, 37^Thr^41^) was increased in the 4‐month‐old d‐gal and 16‐month‐old d‐gal rats compared with the corresponding NS rats (*P* < 0.01) while total β‐catenin protein expression was markedly decreased (*P* < 0.01) (Fig. 2A). As Fig. 2B shows, the similar results were found in 16‐month‐old rats compared with 4‐month‐old rats. To evaluate whether increased phosphorylation of β‐catenin by GSK3β would influence the translocation of β‐catenin, β‐catenin protein expression in nuclear extracts from the tissues was also detected. The level of nuclear β‐catenin protein was lower in d‐gal rats at the ages of 4 months and 16 months compared to the age‐matched NS rats (*P* < 0.05) (Fig. 2C). Furthermore, nuclear β‐catenin protein expression was significantly decreased with aging compared with the same treatment in rats (NS rats: *P* < 0.05, d‐gal rats: *P* < 0.01) (Fig. 2D). To further confirm the localization and protein expression of β‐catenin in the auditory cortex, an immunofluorescence analysis was performed. As our results showed, β‐catenin was mainly located in the cytoplasm and was decreased in 4‐month‐old d‐gal (*P* < 0.05) and 16‐month‐old d‐gal (*P* < 0.01) rats compared with age‐matched NS rats. Moreover, decreased expression of β‐catenin was observed with aging (*P* < 0.05) (Fig. 3A). Quantitative real‐time (RT)‐PCR analysis confirmed that *β‐catenin* mRNA expression was decreased with aging (Fig. 3B). Taken together, these results suggested downregulation of Wnt/β‐catenin signaling in the auditory cortex with aging.

### Activation of Wnt/β‐catenin signaling by long‐term administration of Licl in the auditory cortex

**Figure 4:**
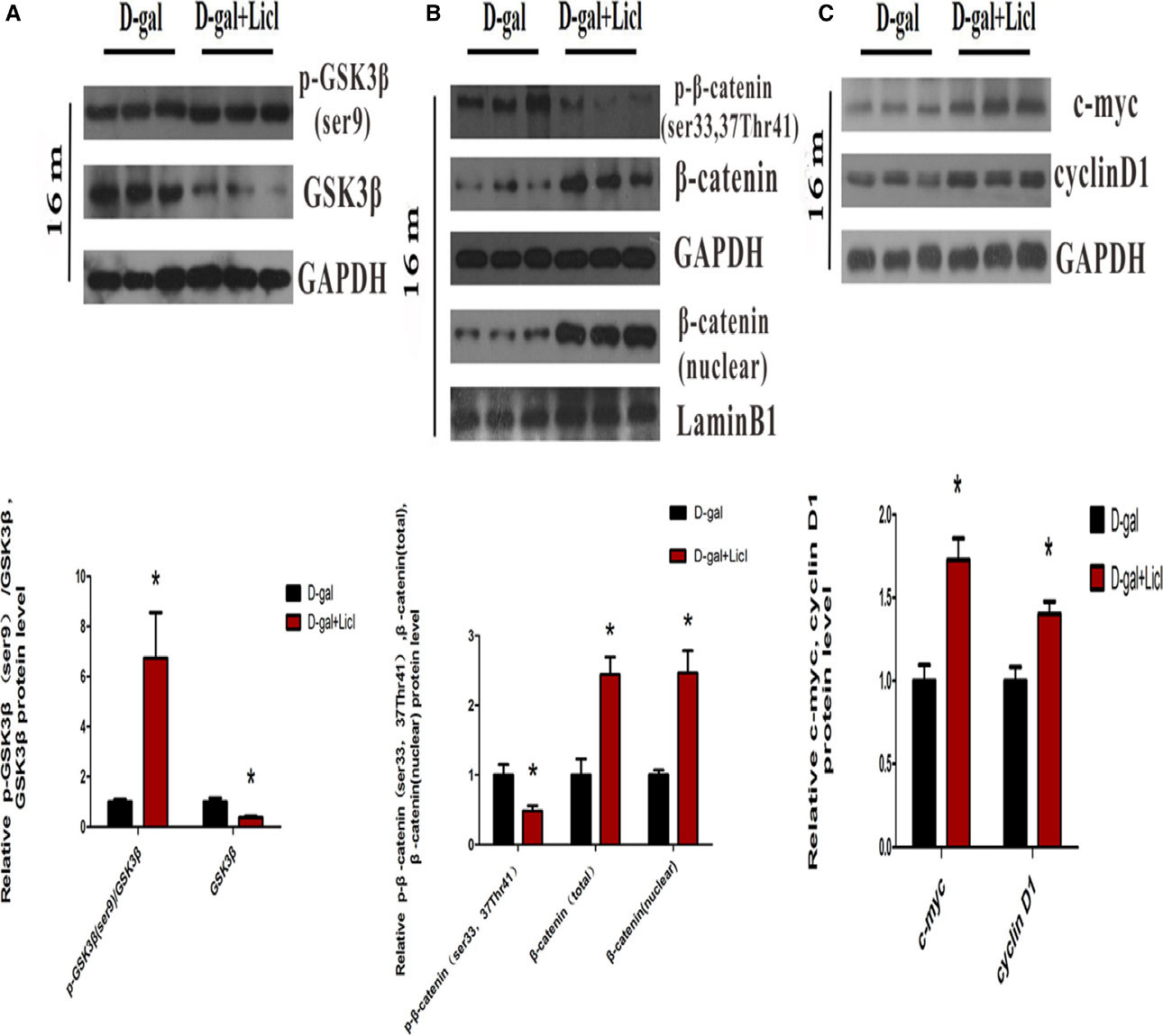
Activation of Wnt/β‐catenin signaling by long‐term administration of Licl in the auditory cortex. (A) Western blot analysis of p‐GSK3β (ser^9^) and GSK3β expression after Licl treatment. (B) Western blot analysis showed p‐β‐catenin (ser^33, 37^Thr^41^), total and nuclear β‐catenin expression after Licl treatment. (C) Western blot analysis of c‐myc and cyclin D1 expression treated with Licl. *P < 0.05, **P < 0.01 vs d‐gal group.

To further investigate the role of canonical Wnt signaling in the auditory cortex during aging, we treated the 15‐month‐old d‐gal rats with the canonical Wnt signaling activator Licl for a month. Western blot analysis was performed to determine whether Licl treatment could activate Wnt signaling in the auditory cortex. Significantly, compared with 16‐month‐old d‐gal rats, we found that Licl treatment resulted in increased in GSK3β phosphorylation at ser^9^ (*P* < 0.05), while there was decreased total GSK3β expression (*P* < 0.05) (Fig. 4A). A significant reduction in phosphorylated β‐catenin at ser^33,37^, Thr^41^ was found after Licl treatment (*P* < 0.05), and Licl was sufficient to enhance β‐catenin stabilization indicated by increased total β‐catenin and alter β‐catenin nuclear localization detected by western blot analysis (Fig. 4B). The mRNA levels of *β‐catenin* showed no marked difference (Fig. 3B), and no obvious nuclear translocation of β‐catenin was detected by immunofluorescence assay (Fig. 3A). A previous study also reported that it is extremely difficult to observe β‐catenin nuclear translocation with an immunocytochemical assay 44. The augmented β‐catenin activity by Licl was further confirmed by the improved expression of β‐catenin downstream molecules, cyclin D1 and c‐myc (*P* < 0.05) (Fig. 4C). The results suggested that Licl treatment efficiently activated Wnt/β‐catenin signaling through inhibition of GSK3β in the auditory cortex.

### Licl attenuated the d‐gal‐induced auditory cortex aging

**Figure 5:**
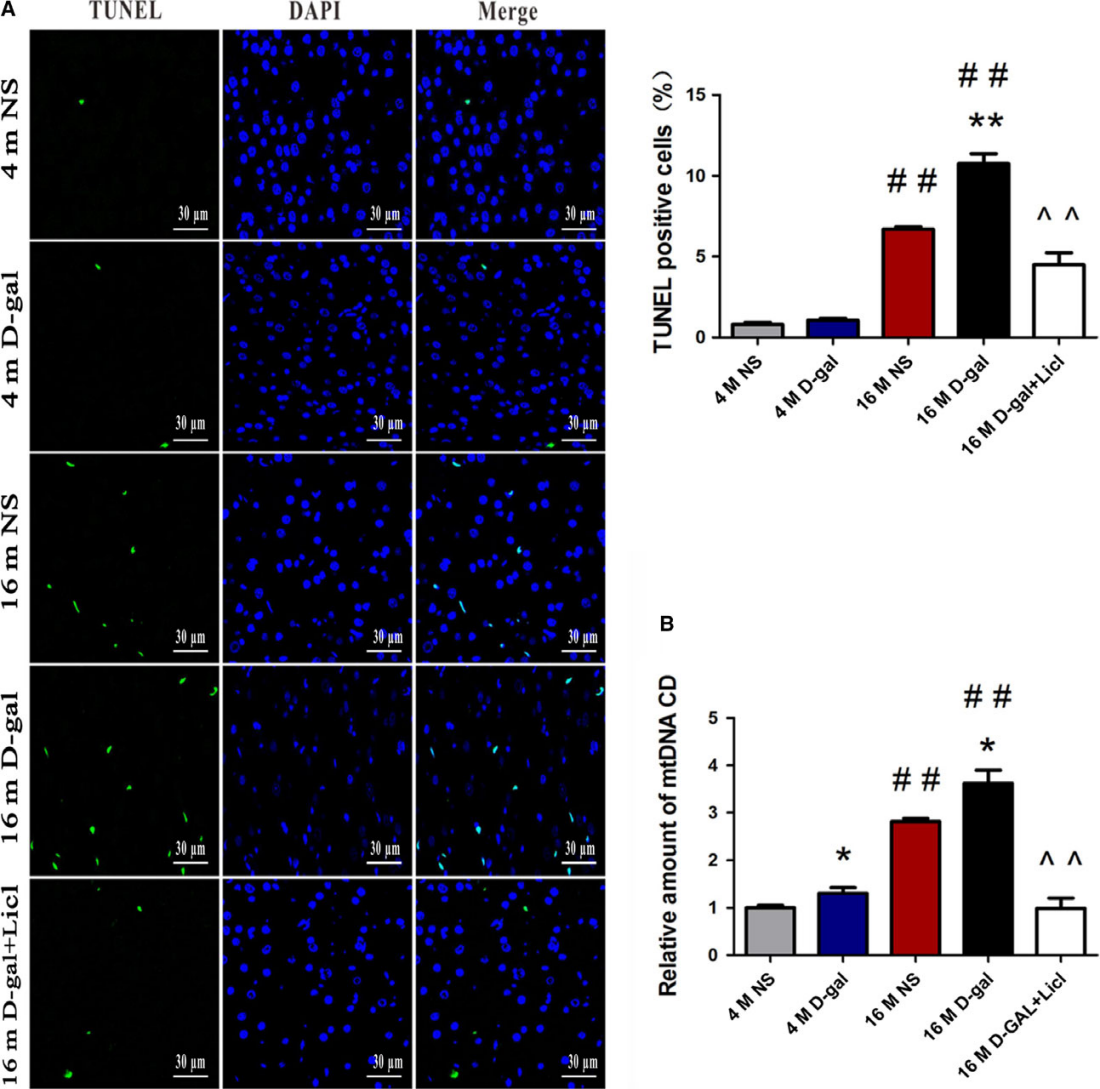
Licl reversed the d‐gal‐induced cell death and CD accumulation in the auditory cortex. (A) TUNEL staining assay showed apoptotic cells in 4‐ and 16‐month‐old NS, d‐gal rats, and 16‐month‐old d‐gal + Licl rats. (B) RT‐PCR analysis of CD levels in 4‐ and 16‐month‐old NS, d‐gal rats, and 16‐month‐old d‐gal + Licl rats. The results were expressed as the mean ± SEM (*n* = 5 for each group). *P < 0.05, **P < 0.01 vs NS group. ##*P* < 0.01 vs 4‐month‐old group. ^^*P* < 0.01 vs 16‐month‐old d‐gal group.

We further investigated whether canonical Wnt signaling could protect against auditory cortex aging. As shown in Fig. 5A, a TUNEL staining assay revealed that the number of TUNEL‐positive cells in the auditory cortex was significantly increased in 16‐month‐old rats compared with 4‐month‐old rats (*P* < 0.01). Furthermore, the number of TUNEL‐positive cells was significantly increased in 16‐month‐old d‐gal rats when compared with the age‐matched NS rats (*P* < 0.01). Notably, while only a few TUNEL‐positive nuclei were found in NS rats and d‐gal rats at the age of 4 months, there was no difference between the two groups. After Licl treatment, we observed that the percentage of apoptotic cells from the auditory cortex was significantly decreased (*P* < 0.01).

The mitochondrial 4834‐bp deletion is also known as the ‘CD’. Previously, our findings revealed that the CD level increased with aging and could be seen as a biomarker for AHL 38, 45. Quantitative PCR (TaqMan probe) analysis was performed to examine the percentage of CD. As shown in Fig. 5B, the CD levels were significantly increased in d‐gal rats at different ages compared with age‐matched control rats (*P* < 0.05). We also demonstrated that the percentage of CD was drastically increased in the 16‐month‐old NS and d‐gal rats compared with 4‐month‐old NS and d‐gal rats (*P* < 0.01). Furthermore, we evaluated the effect of activated canonical Wnt signaling by Licl on the percentage of CD. We found that Licl treatment significantly decreased the CD levels (*P* < 0.01).

**Figure 6:**
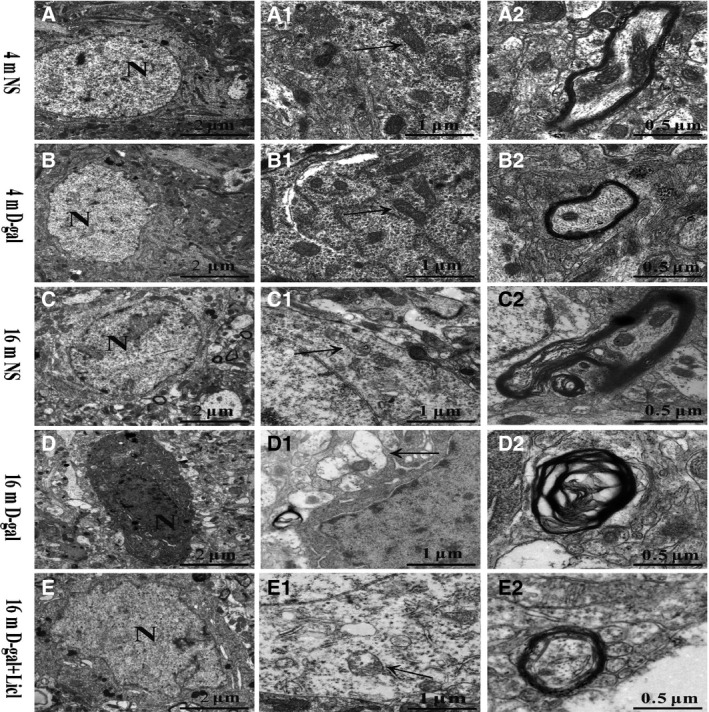
Licl attenuated the d‐gal‐induced neurodegeneration in the auditory cortex. A TEM assay showed ultrastructural changes in the auditory cortex from 4‐ and 16‐month‐old NS, d‐gal rats, and 16‐month‐old d‐gal + Licl rats.

To investigate the effects of aging and activated canonical Wnt signaling on neuron survival, a transmission electron microscopy assay was performed to detect the ultrastructural changes in the auditory cortex. As shown in Fig. 6, in the 4‐month‐old NS group, the neurons of the auditory cortex displayed no obvious ultrastructural changes. An intact nuclear membrane, uniformly dispersed chromatin, normal mitochondria (black arrow), and intact and compact myelin were detected in 4‐month‐old rats, except an irregular nucleus was found in 4‐month‐old d‐gal rats (Fig. 6B). The neurons of the auditory cortex displayed a progressive degeneration in 16‐month‐old rats. An irregular nucleus and condensed chromatin (Fig. 6C,D) were found. The mitochondria were swollen and vacuolated (black arrow in Fig. 6C1,D1), and swollen and disrupted myelin also emerged (Fig. 6C2,D2). Long‐term administration of Licl alleviated d‐gal‐induced neurodegeneration in the auditory cortex, as the ultrastructure of the nucleus, mitochondria, and myelin demonstrated a positive change (Fig. 6E,E1 and E2). Overall, these results demonstrated that the activation of Wnt/β‐catenin signaling by long‐term administration of Licl attenuated d‐gal‐induced auditory cortex aging and neurodegeneration.

### Licl reversed the d‐gal‐induced repression of Bmi1 in the auditory cortex

**Figure 7:**
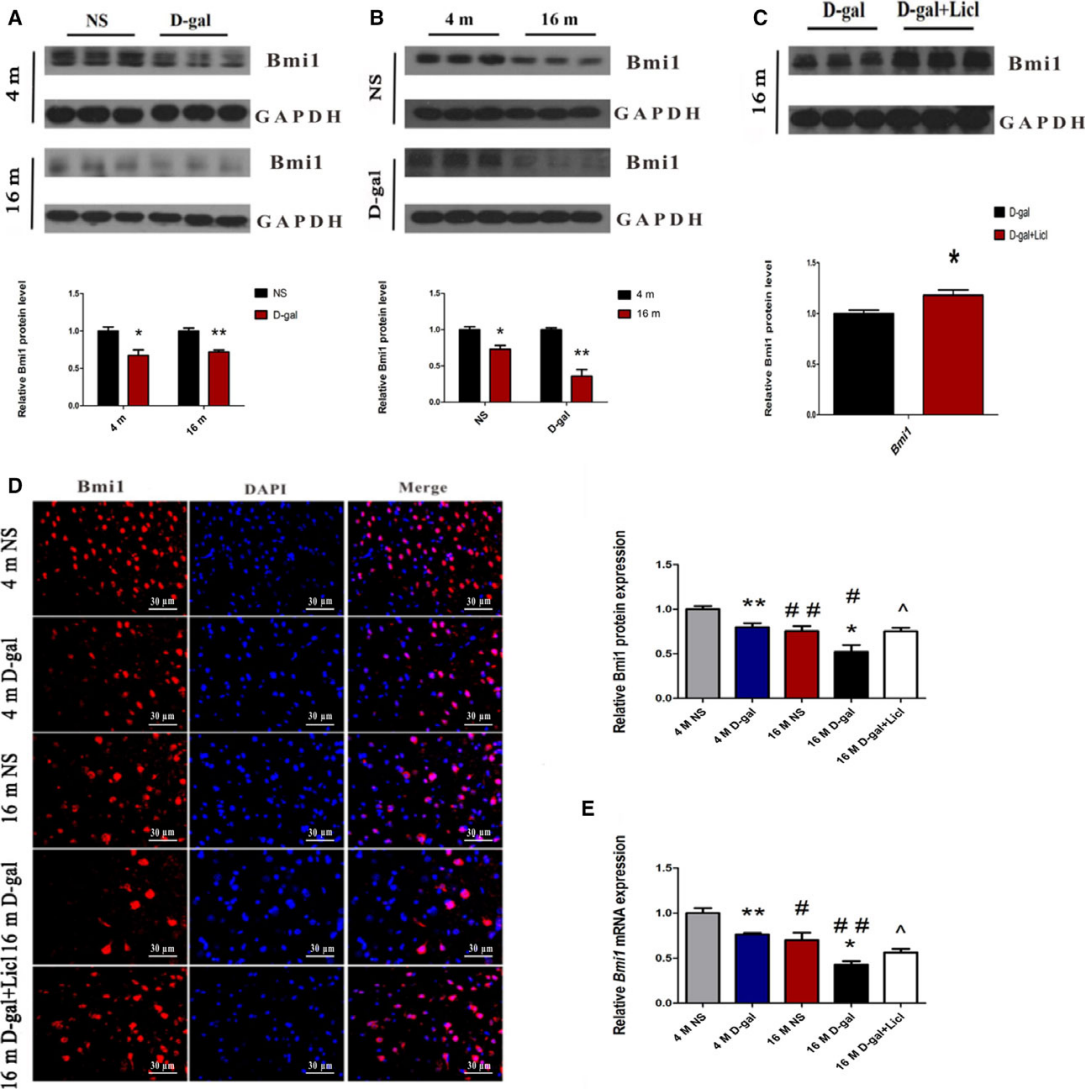
Licl reversed the d‐gal‐induced downregulation of Bmi1 in the auditory cortex. (A) Western blot analysis of Bmi1 expression in d‐gal rats vs NS rats. (B) Western blot analysis of Bmi1 expression in 16‐month‐old rats vs 4‐month‐old rats. (C) Western blot showed Bmi1 expression after treatment with Licl. (D) Immunofluorescence assay of Bmi1 expression in 4‐ and 16‐month‐old NS, d‐gal rats, and 16‐month‐old d‐gal + Licl rats. (E) RT‐PCR analysis of Bmi1 in 4‐ and 16‐month‐old NS, d‐gal rats, and 16‐month‐old d‐gal + Licl rats. *P < 0.05, **P < 0.01 (for A–C). *P < 0.05, **P < 0.01 vs NS group. #*P* < 0.05, ##*P* < 0.01 vs 4‐month‐old group. ^*P* < 0.05 vs 16‐month‐old d‐gal group (for D and E, *n* = 5 for each group).

To further investigate the potential molecular mechanism by which Wnt/β‐catenin signaling is involved in the degeneration of auditory cortex neurons during aging, the expression of Bmi1 was detected. Western blot analysis showed that the Bmi1 protein levels decreased in 4‐month‐old d‐gal (*P* < 0.05) and 16‐month‐old d‐gal (*P* < 0.01) rats compared with the corresponding NS rats (Fig. 7A). Moreover, we found that Bmi1 protein expression was markedly lower in 16‐month‐old NS and d‐gal rats than 4‐month‐old NS and d‐gal rats, respectively (NS rats: *P* < 0.05, d‐gal rats: *P* < 0.01) (Fig. 7B). Next, we investigated whether activated Wnt/β‐catenin signaling would enhance the protein expression of Bmi1. Rats treated with Licl showed greater protein expression compared with 16‐month‐old d‐gal rats (*P* < 0.05) (Fig. 7C). Furthermore, an immunofluorescence assay was performed to detect Bmi1 protein expression. As shown in Fig. 7D, Bmi1 was mainly located in the nuclei and was significantly reduced in d‐gal rats compared with age‐matched NS rats (4‐month‐old rats: *P* < 0.01, 16‐month‐old rats: *P* < 0.05). We also found that Bmi1 expression was dramatically decreased in NS and d‐gal rats at the age of 16‐month‐old compared with the corresponding 4‐month‐old rats (NS rats: *P* < 0.01, d‐gal rats: *P* < 0.05). Real‐time RT‐PCR confirmed the similar changes in *Bmi1* mRNA expression with western blot analysis and an immunofluorescence assay (Fig. 7E).

**Figure 8:**
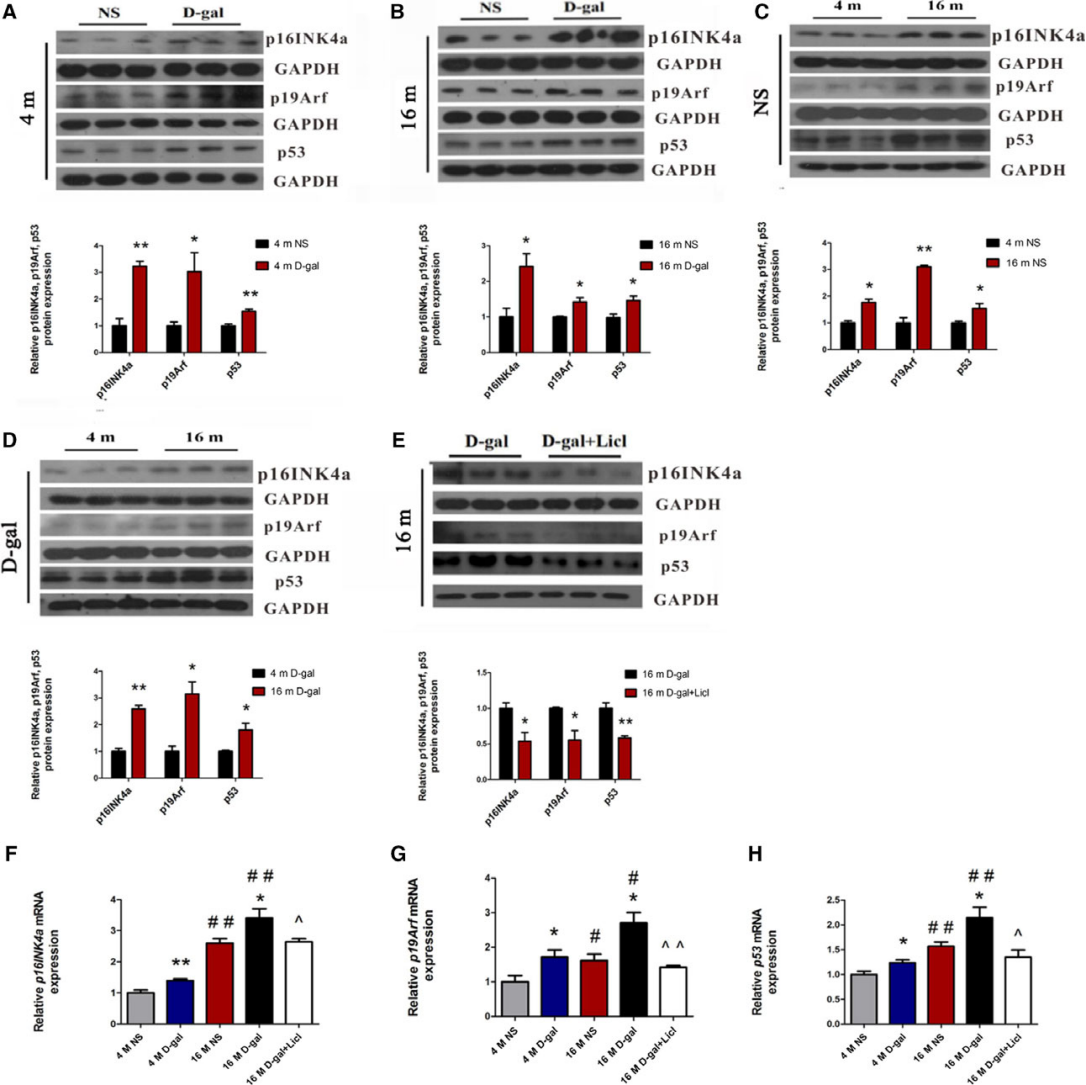
Licl reversed the d‐gal‐induced upregualtion of p16^INK^ ^4a^, p19^Arf^, and p53 in the auditory cortex. (A, B) Western blot analysis of p16^INK^ ^4a^, p19^Arf^, and p53 in d‐gal rats vs NS rats. (C, D) Western blot analysis of p16^INK^ ^4a^, p19^Arf^, and p53 in 16‐month‐old rats vs 4‐month‐old rats. (E) Western blot analysis of p16^INK^ ^4a^, p19^Arf^ and p53 after Licl treatment. (F, G, H) RT‐PCR analysis of p16^INK^ ^4a^, p19^Arf^, and p53 in 4‐month‐old and 16‐month‐old NS, d‐gal rats, and 16‐month‐old d‐gal + Licl rats. *P < 0.05, **P < 0.01 (for A–E). *P < 0.05, **P < 0.01 vs NS group. #*P* < 0.05, ##*P* < 0.01 vs 4‐month‐old group. ^*P* < 0.05, ^^*P* < 0.01 vs 16‐month‐old d‐gal group (for F–H, *n* = 5 for each group).

Bmi1 is known to inhibit the transcription of the INK4a/Arf locus in aging tissues, which encodes the senescence‐associated genes *p16* ^*INK4a*^ and *p19* ^*Arf*^ 35. To evaluate whether altered Bmi1 expression affects the expression of its downstream genes, we investigated the expression of *p16* ^*INK4a*^, *p19* ^*Arf*^, and *p53* in the auditory cortex. As shown in Fig. 8A,B, western blot analysis revealed that p16^INK4a^, p19^Arf^, and p53 expression were significantly increased in 4‐month‐old d‐gal rats compared with 4‐month‐old NS rats (p16^INK4a^: *P* < 0.05, p19^Arf^: *P* < 0.01, p53: *P* < 0.05), increased p16^INK4a^, p19^Arf^, and p53 expression were also found in the 16‐month‐old d‐gal rats compared with 16‐month‐old NS rats (*P* < 0.01). Furthermore, we also compared 4‐month‐old and 16‐month‐old rats, which revealed greater expression of p16^INK4a^, p19^Arf^, and p53 in aged rats (NS rats: p16^INK4a^, *P* < 0.01; p19^Arf^, *P* < 0.05; p53, *P* < 0.01; d‐gal rats: p16^INK4a^, *P* < 0.01; p19^Arf^, *P* < 0.05; p53, *P* < 0.05) (Fig. 8C,D). In contrast, activation of Wnt signaling by Licl reduced the protein levels of p16^INK4a^, p19^Arf^, and p53 (p16^INK4a^: *P* < 0.05, p19^Arf^: *P* < 0.05, p53: *P* < 0.01) (Fig. 8E). Consistently, quantitative RT‐PCR also showed the mRNA expression of the three genes were markedly higher in d‐gal and aged rats. Notably, administration of Licl significantly reduced the mRNA levels of*p16* ^*INK4a*^, *p19* ^*Arf*^, and *p53* compared with 16‐month‐old d‐gal rats (Fig. 8F–H). We concluded that activated canonical Wnt signaling by Licl could reverse the d‐gal‐induced repression of Bmi1 in the auditory cortex.

## Discussion

In this study, we established a mimic aging rat model by chronic systemic exposure of d‐gal. Prior studies showed that an overdose of d‐gal can accelerate aging in animals, which exhibit characteristics that resemble natural aging 46, 47. Moreover, the mimic aging rat model induced by d‐gal is an ideal model for investigating the mechanisms involved in the development of presbycusis 48. Herein, both the effects of d‐gal and aging on the process of auditory cortex degeneration were evaluated. Moreover, long‐term administration of lithium chloride (Licl), an activator of Wnt signaling pathway, was given to the 15‐month‐old d‐gal rats. We demonstrated that an age‐related decrease in Wnt/β‐catenin signaling in the rat auditory cortex and activation of Wnt/β‐catenin signaling by Licl attenuated d‐gal‐induced auditory cortex aging and neurodegeneration in rats. Our data suggest that the attenuation of auditory cortex aging, at least in part, occurs through the enhancement of Bmi1 by β‐catenin.

Our investigations in the underlying molecular mechanisms involved in central presbycusis revealed the dysfunction of Wnt/β‐catenin signaling in the rat auditory cortex during aging. The results showed that both d‐gal and aging induced an increased GSK3β activity, evidenced by a reduction in phosphorylated GSK3β at Ser9. A prior study also reported an age‐associated increase in GSK3β activity in the hippocampus of aged Wistar rats which contributes to the physiopathology of Alzheimer's disease (AD) 49. GSK3β is known to be involved in the regulation of various signaling cellular functions through its ability to phosphorylate a number of substrates 50, 51. As one of the main negative regulators of canonical Wnt signaling, it can inhibit β‐catenin activity by phosphorylated β‐catenin at Ser^33^, Ser^37^, and Thr^41^ 43. β‐catenin, the most important effective molecule in this signaling pathway, is involved in some neurological disorder. It has been reported that reduction of β‐catenin by β‐catenin siRNA contributes to the impairment of neurogenesis in AD progenitor cells 52. Moreover, reduced β‐catenin has been found in the hippocampus of aged Wistar rats 53. Our present data showed that an increase in p‐β‐catenin (ser^33,37^Thr^41^) is accompanied by an enhancement of GSK3β activity in d‐gal and aged rats. Because GSK3β‐mediated phosphorylation of N‐terminal β‐catenin was improved, we predicted that there would be a destabilization of β‐catenin and a reduction in the cytoplasma β‐catenin translocated into the nucleus. As the results showed, the decrease in mRNA and protein of total β‐catenin was also observed in the d‐gal and aged auditory cortex of rats. Moreover, both d‐gal and aging induced a significant decrease in nuclear β‐catenin, which represents the activity of β‐catenin. Taken together, our results revealed an age‐related decline of Wnt/β‐catenin signaling in the auditory cortex during aging. A previous study showed that inhibition of Wnt/β‐catenin signaling causes degeneration of mouse primary hippocampal neurons 54. Our current study showed that accompanied by impaired Wnt/β‐catenin signaling, d‐gal and aged rats displayed greater neuron apoptosis and neurodegeneration in the auditory cortex, except for there was no marked difference between 4‐month‐old NS and d‐gal rats. Considering that d‐gal can be converted into d‐galactitol by aldose reductase, it will accumulate in the cell and cannot be metabolized 55. The possible reason for this striking contrast might be that young auditory cortex neurons have strong resistance to impairment by accumulated d‐galactitol. We indicated that the dysregulation of canonical Wnt signaling could render auditory cortex neurons sensitive to apoptosis and then cause neurodegeneration. Neurodegeneration of the auditory cortex with aging play a key role in the process of the pathogenesis of central presbycusis. Furthermore, as a biomarker for presbycusis, CD was found to be accumulated in d‐gal and aged rats. These results suggested that downregulation of Wnt/β‐catenin signaling in the auditory cortex with aging might participate in the pathogenesis of presbycusis. Aiming to better understand whether canonical Wnt signaling plays a neuroprotective role in the aged auditory cortex, 15‐month‐old d‐gal rats received Licl for 1 month. Licl inhibited GSK3β activity by phosphorylation at serine 9 (ser^9^), resulting in β‐catenin accumulation and nuclear translocation 56. There is no obvious difference in β‐catenin mRNA expression after Licl treatment, this may be due to the fact that Licl regulates β‐catenin in a protein level, and that Licl does not directly affect the transcription process of β‐catenin 13. Besides, it is possible that the β‐catenin mRNA has been degraded when the β‐catenin protein reaches its peak. Studies have also successfully utilized Licl to activate Wnt/β‐catenin signaling both *in vivo* and *in vitro* 57, 58. We showed that activation of Wnt/β‐catenin signaling by Licl attenuated d‐gal‐induced apoptosis and neurodegeneration in the rat auditory cortex. Moreover, chronic Licl treatment dramatically decreased the percentages of CD. Our results suggest that chronic administration of Licl exerted a protective effect against the pathogenesis of central presbycusis mediated by activation of Wnt/β‐catenin signaling.

The participation of Wnt/β‐catenin signaling in the pathogenesis of central presbycusis was partially attributed to the regulation of Bmi1 expression by β‐catenin. Although multiple prosurvival function genes, such as *cyclin D1*,* survivin* and *bcl‐2* regulated by β‐catenin, have been described 59, 60, 61. Little is known about how canonical Wnt signaling affects the tissue aging process. Recently, a study has revealed that β‐catenin enhances Bmi1 expression in a colon cancer cell line 24. Bmi1 plays a vital role in aging, its deficiency has been shown to cause premature aging and contribute to an early onset of brain aging 28, 29. In addition, Bmi1 extended the lifespan of normal human oral keratinocytes, and silencing Bmi1 enhances the senescence of human gastric cancer cells 62, 63. In line with the downregulation of β‐catenin activity, the mRNA and protein levels of Bmi1 were dramatically decreased after d‐gal administration, and decreased expression of Bmi1 was also found in the aged rat auditory cortex. The antiaging role of Bmi1 is mainly due to its ability to inhibit the transcription of the *Ink4a/Arf* locus, and then suppress thep16^INK4a^ and p19^Arf^/p53 pathways 35. In this context, d‐gal‐ and aging‐induced downregulation of Bmi1 expression might contribute to the increased transcription of p16^INK4a^, which could inhibit cell cycle progression, and increased p16^INK4a^ unbalances tissue homeostasis during aging 64. Moreover, the p19^Arf^/p53 pathway is a main signal transduction pathway that controls cell aging processes 65. This pathway was found to be upregulated in the d‐gal and aged rat auditory cortex. The results indicated that the weaken Bmi1 expression increases the p19^Arf^/p53 pathway activity and initiates apoptosis. A study also showed that *Bmi1* knockdown neurons have increased p19^Arf^ and p53 expression, and the study showed that p53 mediates most of the apoptotic phenotypes 25. Our results further indicated that β‐catenin could regulate Bmi1 expression in the auditory cortex during aging, because we found higher Bmi1 expression followed by an augmented β‐catenin activity after Licl administration. Moreover, in agreement with the alleviation of d‐gal‐induced aging features by activated Wnt/β‐catenin signaling, Licl significantly decreased p16^INK4a^, p19^Arf^, and p53 expression levels in rat auditory cortex. The more recent study reported that activation of Wnt/β‐catenin signaling by Licl provides neuroprotection against hypoglycemia‐induced apoptosis in neuron PC12 cells 66. In addition, Licl has been revealed to reduce cell death by modulating p53 and bcl‐2 expression, which is related to the initiation of the apoptotic processes 67, 68. In this research, our results suggested that long‐term administration of Licl increased Bmi1 expression, and then Bmi1 reduced these age‐associated genes, and subsequently reversed d‐gal‐induced phenotype of central presbycusis. Although the participation of other mechanisms cannot be excluded, our finding supports the idea that activation of Wnt/β‐catenin signaling by Licl attenuated d‐gal‐induced auditory cortex aging and neurodegeneration partially by enhancement of Bmi1 expression. As the effects of chronic systemic exposure to d‐gal on aging features in the auditory cortex resemble natural aging, our study sheds light on a promising intervention for the delay, even reversal, of the natural aging process of the auditory cortex by activation of Wnt/β‐catenin signaling. However, there was no substantial change in cell apoptosis and ultrastructural morphology in the auditory cortex between 4‐month‐old NS and 4‐month‐old d‐gal rats. Our previous studies also showed this phenomenon 39, 45. Furthermore, it is hard to observe a difference in cell apoptosis and ultrastructural morphology, and even the molecular expression lacked statistically significant differences after Licl treatment in the auditory cortex of young d‐gal rats (date not shown), therefore, we paid more attention to the effects of activated Wnt signaling on aged d‐gal rats. To fully understand the protective mechanism of Wnt signaling against the pathogenesis of presbycusis, future works should be carried out with more complete solutions using multiple pharmacological and genetic approaches.

In conclusion, our results revealed that mimetic aging rats induced by d‐gal had an age‐related decrease in Wnt/β‐catenin signaling accompanied by downregulation of Bmi1, which is involved in neurodegeneration of the auditory cortex. In addition, activation of Wnt/β‐catenin signaling by long‐term treatment with Licl reversed neurodegeneration induced by d‐gal, which might occur through modulation of the expression of Bmi1. These findings offer novel evidence for the protective effects against aging of Wnt/β‐catenin signaling in age‐related auditory cortex degeneration and provide a potential therapeutic direction for presbycusis.

## Materials and methods

### Animal procedures

One hundred and twenty male Sprague–Dawley rats (4 weeks old) were purchased from the Experimental Animal Center of Tongji Medical College, Huazhong University of Science and Technology. After acclimation for 4 weeks, the rats (2 months old) were randomly divided into the following three groups: a control group, a d‐gal (Sigma Chemical, St. Louis, MO, USA) group, and a d‐gal + Licl (Sigma Chemical) group. The rats in the d‐gal group were injected subcutaneously with d‐gal (500 mg·kg^−1^·day^−1^) for 8 weeks while the rats in the control group were injected with the same volume of vehicle (0.9% saline) for 8 weeks. On the basis of the d‐gal group (15 months old), the rats in the d‐gal + Licl group received a daily lithium chloride (60 mg·kg^−1^) injection subcutaneously for a total of 30 days. The Licl treatment for the d‐gal + Licl group was terminated on the day the rats were killed. Both the control and d‐gal groups were divided into two age subgroups: 4‐month‐old (just after the injection) and 16‐month‐old (12 months after the last injection). All rats were individually housed in standard polycarbonate cages in a temperature‐controlled (24 ± 2 °C) and light‐controlled environment with a 12‐h light⁄dark cycle and were provided standard rodent chow and water.

All experimental procedures were performed in accordance with the National Institute of Health Guide for the Care and Use of Laboratory Animals. The protocol was under the supervision of the Committee on the Ethics of Animal Experiments of Huazhong University of Science and Technology.

### Protein extraction and western blot analysis

Total and nuclear protein from rat auditory cortex tissues were extracted using the Total Protein Extraction Kit and the Nuclear Protein Extraction Kit, respectively (Beyotime, Haimen, China). For western blot on total extracts, auditory cortex tissues were washed with cold PBS, and were homogenized in lysis buffer using a homogenizer on the ice for 30 min. The samples were then centrifuged at 10 000 ***g*** for 10 min at 4 °C. The supernatant containing total proteins were collected. For western blot on nuclear extracts, auditory cortex tissues were homogenized in lysis buffer on the ice and centrifuged at 12 000 ***g*** at 4 °C for 5 min. The supernatants were collected as cytoplasmic extracts, and the sediments were resuspended in the lysis buffer on ice for 10 min. After the addition of 10% NP‐40, samples were vigorously vortexed three times, then centrifuged at 12 000 ***g*** for 5 min. The supernatants were discarded and the sediments were resuspended in the extraction buffer on ice for 30 min, samples were vigorously vortexed five times, then centrifuged at 12 000 ***g*** at 4 °C for 15 min. The supernatant containing nuclear proteins were collected. Protein concentrations were determined with the BCA Protein Assay Kit (Beyotime). An equal amount of protein lysate (20 µg) was loaded onto 10% SDS/PAGE gels for electrophoresis. After appropriate separation, the proteins were transferred onto a PVDF membrane blocked for 1 h in 5% nonfat dry milk diluted in Tris‐buffered saline( 0.1 m) added to 0.1% Tween‐20 (TBST), and then incubated overnight at 4 °C with the appropriate dilution of the following primary antibodies: anti‐β‐catenin (1 : 1000, Cell Signaling Technology, Danvers, MA, USA), anti‐p‐β‐catenin Ser^33,37^Thr^41^ (1 : 1000, Cell Signaling Technology), anti‐GSK3β (1 : 2000, Wanleibio, Shenyang, China), anti‐p‐GSK3β Ser^9^ (1 : 1000, Cell Signaling Technology), anti‐Bmi1 (1 : 1000, Abcam, Cambridge, MA, USA), anti‐c‐myc (1 : 1000, Abcam), anti‐cyclinD1 (1 : 1000, Abcam), anti‐p16^Ink4a^ (1 : 1000, Proteintech, Wuhan, China), anti‐p19^Arf^ (1 : 200, Santa Cruz Biotechnology, Santa Cruz, CA, USA), anti‐p53 (1 : 200, Santa Cruz Biotechnology), anti‐GAPDH (1 : 2000, AntGene, Wuhan, China), and anti‐LaminB1 (1 : 2000, Wanleibio, Shenyang, China). After washing with TBST three times, the membranes were incubated with the appropriate secondary antibody at room temperature for 1 h, followed by washing of the membrane with TBS‐T three times. Immunoblots were visualized with an ECL Western Blotting Kit (Beyotime). GAPDH was detected as a loading control for total protein and LaminB1 was detected as a loading control for nuclear protein.

### RNA isolation and quantitative real‐time PCR

Total RNA from fresh auditory cortex tissues was extracted using Trizol reagent (Invitrogen, Carlsbad, CA, USA), according to the manufacturer's instructions. cDNA was reverse transcribed using the Prime Script RT reagent Kit (Takara Bio, Otsu, Japan). The concentrations and purification of the RNA and cDNA of each sample were analyzed using the Gene Quant Pro DNA/RNA Calculator (BioChrom, Cambridge, UK). The cDNA samples were stored at −20 °C until further use. Real‐time quantitative PCR was performed by applying real‐time SYBR Green PCR technology with the use of the lightcycle 480 RT‐PCR system (Roche Diagnostics Ltd, Rotkreuz, Switzerland). The PCR reactions were performed in a volume of 20 μL and contained 2 μL of diluted cDNA, 0.8 μL of forward and reverse primers, 6.4 μL of DEPC water (Biosharp, Hefei, China), and 10 μL of SYBR Green Real‐time PCR Master Mix Kit (Takara Bio). Primers for the qPCR (listed in Table 1) used for amplification were designed using primer premier 5.0 software (Premier Biosoft International, Palo Alto, CA, USA).

**Table 1:** The nucleotide sequences of the primers that were used in the real‐time PCR analysis with SYBR Green

Gene	Sequence (5′‐3′)
*β‐catenin*	Forward, 5′‐TCTTGGCTATTACGACAGACT‐3′
	Reverse, 5′‐CCACCCACTTGGCACA‐3′
*Bmi1*	Forward, 5′‐AAGGAGGAGGTGAATGAT‐3′
	Reverse, 5′‐AGGTGTAAATGTAGGCAAT‐3′
*p16* ^*Ink4a*^	Forward, 5′‐TCCGAGAGGAAGGCGAACTC‐3′
	Reverse, 5′‐GCTGCCCTGGCTAGTCTATCTG‐3′
*p19* ^*Arf*^	Forward, 5′‐ACCCCAAGTGAGGGTTTTCT‐3′
	Reverse, 5′‐GATCCTCTCTGGCCTCAACA‐3′
*p53*	Forward, 5′‐CATCTTCCGTCCCTTCTCAA‐3′
	Reverse, 5′‐AGCGTCTCACGACCTCAGTC‐3′
*GAPDH*	Forward, 5′‐AGCCGTTCGGAGGATTATTCG‐3′
	Reverse, 5′‐CTTCTCCTCAGCAGCCAGAG‐3′

The amplification protocol was as follows: 1 cycle at 95 °C for 5 min, 45 cycles at 95 °C for 10 s, and 52 °C for 20 s, then 72 °C for 20 s, and an extension at 72 °C for 5 min. The relative mRNA expression of each group was analyzed using the 2^−ΔΔct^ method.

### Immunofluorescence

Immunofluorescence was used to detect protein expression of β‐catenin and Bmi1 in the auditory cortex. After being deeply anesthetized, the rats were transcardially perfused with a 0.9% normal saline wash, following by a 4% paraformaldehyde fixative. When perfusion was finished, the brains were removed and postfixed in 4% paraformaldehyde overnight at 4 °C. After rinsing with distilled water, all brains were dehydrated through graded concentrations of an ethanol series, cleared in xylene, and then immersed in paraffin. Following deparaffinization, rehydration, and antigen retrieval according to standard protocols, the samples were blocked with donkey serum albumin for 1 h at room temperature and then incubated overnight with anti‐β‐catenin (1 : 100, Cell Signaling Technology) and anti‐Bmi1 (1 : 200, Abcam) at 4 °C in a humidified chamber. After three washes in PBS, sections were incubated with secondary fluorescently tagged antibodies for 1 h at room temperature. Slides were mounted on cover slips with a DAPI solution; then, a laser‐scanning confocal microscope (LSCM) (Nikon, Tokyo, Japan) was using to observe the samples.

### DNA extraction and quantitation of the mtDNA common deletion

Total DNA was extracted from the auditory cortex with the Genomic DNA Purification Kit (Tiangen Biotech Co., Ltd, Beijing, China) according to the manufacturer's instructions. The Gene Quant ProDNA/RNA Calculator (BioChrom) was used to measure the DNA concentration of each sample. The percentages of CD were measured with a TaqMan quantitative real‐time PCR assay. The copy number of the mitochondrial D‐loop region was used as a measure of the total amount of mtDNA in each sample. The PCR primer and probe sequences for the D‐loop region and mtDNA CD have been previously described 69. PCR amplification was performed on a lightcycle 480 RT‐PCR system (Roche Diagnostics Ltd) in a 20 μL reaction mixture containing: 10 μL of TaqMan PCR Master Mix Kit (Takara Bio), 6.4 μL of DEPC water (Biosharp), 0.8 μL of each probe (10 mm), 0.4 μL of each reverse and forward primer (10 mm), and 2 μL of the DNA sample. The amplification conditions were as follows: a 2‐min initial step at 50 °C, then 10 min at 95 °C, 40 cycles of 15 s each at 95 °C, and finally 1 min at 60 °C. The abundance of CD was calculated from the cycle threshold (CT) value, which represents the PCR cycle number at which the fluorescence signal reaches a significant increase. The measurement of relative abundance was measured by the difference in CT values (ΔCT). ΔCT (CT_deletion_−CT_dloop_) was used to calculate the abundance of the mtDNA CD. The relative expression was calculated by the 2^−ΔΔct^ method, which reflects the difference in the deletions between each group.

### TUNEL staining

To determine the amount of cell death and cleavage of DNA, a terminal deoxynucleotidyl transferase‐mediated deoxyuridine 5′‐triphosphate nick‐end labeling (TUNEL) assay (Roche Diagnostics, Mannheim, Germany) was used. Following deparaffinization, rehydration and antigen retrieval according to standard protocols, the sections were incubated of 50 mL of the TUNEL assay solution at 37 °C for 60 min in the dark. After three washes in PBS, sections were stained with DAPI for nuclei staining. Labeled cells were detected with a laser‐scanning confocal microscope (LSCM) (Nikon). The percent of TUNEL‐positive cells was defined as number of TUNEL‐positive cells/number of all cells.

### Transmission electron microscopy

The rats were deeply anesthetized, then perfused transcardially with a quick wash of 0.9% oxygenated saline, which was followed by 2.5% glutaraldehyde in 0.1 m phosphate buffer (pH 7.2). The auditory cortex tissues dissected from the skull were fixed with 2.5% glutaraldehyde in 0.1 m phosphate buffer (PH = 7.2) for 12 h at 4 °C. After that, the auditory cortex tissues were postfixed with 1% cacodylate‐buffered osmium tetroxide at room temperature for 2 h. The tissues were gradually dehydrated with a series of ethanol from 30% to 70% and embedded in Epon‐Araldite. A series of ultrathin sections were cut with a diamond knife, mounted on copper grids, stained with uranyl acetate, and then stained with 4% uranyl acetate and 0.4% lead citrate. The ultrastructure of the stained sections was observed with a Transmission Electron Microscope (FEITecnaiG212, Phillips, Amsterdam, the Netherlands).

### Statistical analysis

All of the results are presented as the mean ± SEM. Statistical analysis was performed using spss 13.0 software (IBM, Armonk, NY, USA). One‐way ANOVA was used and a difference was considered statistically significant at *P* < 0.05.

## Author contributions

MYX, XYZ, and WK conceived and designed the experiments. MYX, XYZ, QLH, HYS, CS, JY, and CH performed the experiments. XYZ, QLH, and SY provided technical advice to the experiment procedures. MYX analyzed data and drafted the paper. XYZ, HYS, WJK, and WK participated in the revision of the manuscript. WJK and WK provided the conceptual framework for the study, directed and coordinated the project. All authors approved the final version of the manuscript.

## References

[1] Gates GA and Mills JH (2005) Presbycusis. Lancet 366, 1111–1120. 16182900 10.1016/S0140-6736(05)67423-5

[2] Someya S , Xu J , Kondo K , Ding D , Salvi RJ , Yamasoba T , Rabinovitch PS , Weindruch R , Leeuwenburgh C , Tanokura M *et al* (2009) Age‐related hearing loss in C57BL/6J mice is mediated by Bak‐dependent mitochondrial apoptosis. Proc Natl Acad Sci USA 106, 19432–19437. 19901338 10.1073/pnas.0908786106 PMC2780799

[3] Bielefeld EC , Tanaka C , Chen GD and Henderson D (2010) Age‐related hearing loss: is it a preventable condition? Hear Res 264, 98–107. 19735708 10.1016/j.heares.2009.09.001 PMC2868117

[4] Gates GA (2012) Central presbycusis: an emerging view. Otolaryngol–Head Neck Surg 147, 1–2. 22535914 10.1177/0194599812446282

[5] Ouda L , Profant O and Syka J (2015) Age‐related changes in the central auditory system. Cell Tissue Res 361, 337–358. 25630878 10.1007/s00441-014-2107-2

[6] Bance M (2007) Hearing and aging. Canad Med Associat J 176, 925–927. 10.1503/cmaj.070007 PMC1828179 17389438

[7] Briley PM and Summerfield AQ (2014) Age‐related deterioration of the representation of space in human auditory cortex. Neurobiol Aging 35, 633–644. 24094582 10.1016/j.neurobiolaging.2013.08.033

[8] Costa M , Lepore F , Prevost F and Guillemot JP (2016) Effects of aging on peripheral and central auditory processing in rats. Eur J Neuorsci 44, 2084–2094. 10.1111/ejn.13302 27306460

[9] Profant O , Balogova Z , Dezortova M , Wagnerova D , Hajek M and Syka J (2013) Metabolic changes in the auditory cortex in presbycusis demonstrated by MR spectroscopy. Exp Gerontol 48, 795–800. 23648586 10.1016/j.exger.2013.04.012

[10] Kim W , Kim M and Jho EH (2013) Wnt/beta‐catenin signalling: from plasma membrane to nucleus. Biochem J 450, 9–21. 23343194 10.1042/BJ20121284

[11] MacDonald BT , Tamai K and He X (2009) Wnt/beta‐catenin signaling: components, mechanisms, and diseases. Dev Cell 17, 9–26. 19619488 10.1016/j.devcel.2009.06.016 PMC2861485

[12] Ye X , Zerlanko B , Kennedy A , Banumathy G , Zhang R and Adams PD (2007) Downregulation of Wnt signaling is a trigger for formation of facultative heterochromatin and onset of cell senescence in primary human cells. Mol Cell 27, 183–196. 17643369 10.1016/j.molcel.2007.05.034 PMC2698096

[13] Clement‐Lacroix P , Ai M , Morvan F , Roman‐Roman S , Vayssiere B , Belleville C , Estrera K , Warman ML , Baron R and Rawadi G (2005) Lrp5‐independent activation of Wnt signaling by lithium chloride increases bone formation and bone mass in mice. Proc Natl Acad Sci USA 102, 17406–17411. 16293698 10.1073/pnas.0505259102 PMC1297659

[14] Moon RT , Kohn AD , De Ferrari GV and Kaykas A (2004) WNT and beta‐catenin signalling: diseases and therapies. Nat Rev Genet 5, 691–701. 15372092 10.1038/nrg1427

[15] Reya T and Clevers H (2005) Wnt signalling in stem cells and cancer. Nature 434, 843–850. 15829953 10.1038/nature03319

[16] Iyer S , Han L , Bartell SM , Kim HN , Gubrij I , de Cabo R , O'Brien CA , Manolagas SC and Almeida M (2014) Sirtuin1 (Sirt1) promotes cortical bone formation by preventing beta‐catenin sequestration by FoxO transcription factors in osteoblast progenitors. J Biol Chem 289, 24069–24078. 25002589 10.1074/jbc.M114.561803 PMC4148840

[17] Manolagas SC and Almeida M (2007) Gone with the Wnts: beta‐catenin, T‐cell factor, forkhead box O, and oxidative stress in age‐dependent diseases of bone, lipid, and glucose metabolism. Mol Endocrinol 21, 2605–2614. 17622581 10.1210/me.2007-0259

[18] Salins P , Shawesh S , He Y , Dibrov A , Kashour T , Arthur G and Amara F (2007) Lovastatin protects human neurons against Abeta‐induced toxicity and causes activation of beta‐catenin‐TCF/LEF signaling. Neurosci Lett 412, 211–216. 17234346 10.1016/j.neulet.2006.07.045

[19] Kuo BR , Baldwin EM , Layman WS , Taketo MM and Zuo J (2015) In vivo cochlear hair cell generation and survival by coactivation of beta‐catenin and Atoh1. J Neurosci 35, 10786–10798. 26224861 10.1523/JNEUROSCI.0967-15.2015 PMC4518053

[20] Munnamalai V and Fekete DM (2013) Wnt signaling during cochlear development. Semin Cell Dev Biol 24, 480–489. 23548730 10.1016/j.semcdb.2013.03.008 PMC3690158

[21] Wang T , Chai R , Kim GS , Pham N , Jansson L , Nguyen DH , Kuo B , May LA , Zuo J , Cunningham LL *et al* (2015) Lgr5+ cells regenerate hair cells via proliferation and direct transdifferentiation in damaged neonatal mouse utricle. Nat Commun 6, 6613. 25849379 10.1038/ncomms7613 PMC4391285

[22] Gonzalez‐Valdes I , Hidalgo I , Bujarrabal A , Lara‐Pezzi E , Padron‐Barthe L , Garcia‐Pavia P , Gomez‐del Arco P , Redondo JM , Ruiz‐Cabello JM , Jimenez‐Borreguero LJ *et al* (2015) Bmi1 limits dilated cardiomyopathy and heart failure by inhibiting cardiac senescence. Nat Commun 6, 6473. 25751743 10.1038/ncomms7473 PMC5603726

[23] Xu F , Yang R , Wu L , He Q , Zhang Z , Zhang Q , Yang Y , Guo J , Chang C and Li X (2012) Overexpression of BMI1 confers clonal cells resistance to apoptosis and contributes to adverse prognosis in myelodysplastic syndrome. Cancer Lett 317, 33–40. 22120066 10.1016/j.canlet.2011.11.012

[24] Yu T , Chen X , Zhang W , Colon D , Shi J , Napier D , Rychahou P , Lu W , Lee EY , Weiss HL *et al* (2012) Regulation of the potential marker for intestinal cells, Bmi1, by beta‐catenin and the zinc finger protein KLF4: implications for colon cancer. J Biol Chem 287, 3760–3768. 22170051 10.1074/jbc.M111.316349 PMC3281718

[25] Chatoo W , Abdouh M , David J , Champagne MP , Ferreira J , Rodier F and Bernier G (2009) The polycomb group gene Bmi1 regulates antioxidant defenses in neurons by repressing p53 pro‐oxidant activity. J Neurosci 29, 529–542. 19144853 10.1523/JNEUROSCI.5303-08.2009 PMC2744209

[26] Van der Lugt NM , Domen J , Linders K , van Roon M , Robanus‐Maandag E , te Riele H , van der Valk M , Deschamps J , Sofroniew M , van Lohuizen M *et al* (1994) Posterior transformation, neurological abnormalities, and severe hematopoietic defects in mice with a targeted deletion of the bmi‐1 proto‐oncogene. Genes Dev 8, 757–769. 7926765 10.1101/gad.8.7.757

[27] Abdouh M , Chatoo W , El Hajjar J , David J , Ferreira J and Bernier G (2012) Bmi1 is down‐regulated in the aging brain and displays antioxidant and protective activities in neurons. PLoS One 7, e31870. 22384090 10.1371/journal.pone.0031870 PMC3285640

[28] Cao G , Gu M , Zhu M , Gao J , Yin Y , Marshall C , Xiao M , Ding J and Miao D (2012) Bmi‐1 absence causes premature brain degeneration. PLoS One 7, e32015. 22363787 10.1371/journal.pone.0032015 PMC3282795

[29] Gu M , Shen L , Bai L , Gao J , Marshall C , Wu T , Ding J , Miao D and Xiao M (2014) Heterozygous knockout of the Bmi‐1 gene causes an early onset of phenotypes associated with brain aging. Age 36, 129–139. 23771506 10.1007/s11357-013-9552-9 PMC3889899

[30] Biehs B , Hu JK , Strauli NB , Sangiorgi E , Jung H , Heber RP , Ho S , Goodwin AF , Dasen JS , Capecchi MR *et al* (2013) BMI1 represses Ink4a/Arf and Hox genes to regulate stem cells in the rodent incisor. Nat Cell Biol 15, 846–852. 23728424 10.1038/ncb2766 PMC3735916

[31] Bracken AP , Kleine‐Kohlbrecher D , Dietrich N , Pasini D , Gargiulo G , Beekman C , Theilgaard‐Monch K , Minucci S , Porse BT , Marine JC *et al* (2007) The Polycomb group proteins bind throughout the INK4A‐ARF locus and are disassociated in senescent cells. Genes Dev 21, 525–530. 17344414 10.1101/gad.415507 PMC1820894

[32] Dhawan S , Tschen SI and Bhushan A (2009) Bmi‐1 regulates the Ink4a/Arf locus to control pancreatic beta‐cell proliferation. Genes Dev 23, 906–911. 19390085 10.1101/gad.1742609 PMC2675870

[33] Molofsky AV , He S , Bydon M , Morrison SJ and Pardal R (2005) Bmi‐1 promotes neural stem cell self‐renewal and neural development but not mouse growth and survival by repressing the p16Ink4a and p19Arf senescence pathways. Genes Dev 19, 1432–1437. 15964994 10.1101/gad.1299505 PMC1151659

[34] Molofsky AV , Slutsky SG , Joseph NM , He S , Pardal R , Krishnamurthy J , Sharpless NE and Morrison SJ (2006) Increasing p16INK4a expression decreases forebrain progenitors and neurogenesis during ageing. Nature 443, 448–452. 16957738 10.1038/nature05091 PMC2586960

[35] Sharpless NE (2004) Ink4a/Arf links senescence and aging. Exp Gerontol 39, 1751–1759. 15582292 10.1016/j.exger.2004.06.025

[36] Meissner C , Bruse P , Mohamed SA , Schulz A , Warnk H , Storm T and Oehmichen M (2008) The 4977 bp deletion of mitochondrial DNA in human skeletal muscle, heart and different areas of the brain: a useful biomarker or more? Exp Gerontol 43, 645–652. 18439778 10.1016/j.exger.2008.03.004

[37] Nicklas JA , Brooks EM , Hunter TC , Single R and Branda RF (2004) Development of a quantitative PCR (TaqMan) assay for relative mitochondrial DNA copy number and the common mitochondrial DNA deletion in the rat. Environ Mol Mutagen 44, 313–320. 15476199 10.1002/em.20050

[38] Kong WJ , Wang Y , Wang Q , Hu YJ , Han YC and Liu J (2006) The relation between D‐galactose injection and mitochondrial DNA 4834 bp deletion mutation. Exp Gerontol 41, 628–634. 16716550 10.1016/j.exger.2006.04.008

[39] Sun HY , Hu YJ , Zhao XY , Zhong Y , Zeng LL , Chen XB , Yuan J , Wu J , Sun Y , Kong W *et al* (2015) Age‐related changes in mitochondrial antioxidant enzyme Trx2 and TXNIP‐Trx2‐ASK1 signal pathways in the auditory cortex of a mimetic aging rat model: changes to Trx2 in the auditory cortex. FEBS J 282, 2758–2774. 25996168 10.1111/febs.13324

[40] Markaryan A , Nelson EG and Hinojosa R (2009) Quantification of the mitochondrial DNA common deletion in presbycusis. Laryngoscope 119, 1184–1189. 19358252 10.1002/lary.20218

[41] McManus EJ , Sakamoto K , Armit LJ , Ronaldson L , Shpiro N , Marquez R and Alessi DR (2005) Role that phosphorylation of GSK3 plays in insulin and Wnt signalling defined by knockin analysis. EMBO J 24, 1571–1583. 15791206 10.1038/sj.emboj.7600633 PMC1142569

[42] Al‐Fageeh M , Li Q , Dashwood WM , Myzak MC and Dashwood RH (2004) Phosphorylation and ubiquitination of oncogenic mutants of β‐catenin containing substitutions at Asp32. Oncogene 23, 4839–4846. 15064718 10.1038/sj.onc.1207634 PMC2267883

[43] Castellone MD , De Falco V , Rao DM , Bellelli R , Muthu M , Basolo F , Fusco A , Gutkind JS and Santoro M (2009) The beta‐catenin axis integrates multiple signals downstream from RET/papillary thyroid carcinoma leading to cell proliferation. Can Res 69, 1867–1876. 10.1158/0008-5472.CAN-08-1982 PMC2746012 19223551

[44] Jacques BE , Montgomery WHT , Uribe PM , Yatteau A , Asuncion JD , Resendiz G , Matsui JI and Dabdoub A (2014) The role of Wnt/beta‐catenin signaling in proliferation and regeneration of the developing basilar papilla and lateral line. Develop Neurobiol 74, 438–456. 10.1002/dneu.22134 24115534

[45] Zeng L , Yang Y , Hu Y , Sun Y , Du Z , Xie Z , Zhou T and Kong W (2014) Age‐related decrease in the mitochondrial sirtuin deacetylase Sirt3 expression associated with ROS accumulation in the auditory cortex of the mimetic aging rat model. PLoS One 9, e88019. 24505357 10.1371/journal.pone.0088019 PMC3913718

[46] Lu J , Zheng YL , Luo L , Wu DM , Sun DX and Feng YJ (2006) Quercetin reverses D‐galactose induced neurotoxicity in mouse brain. Behav Brain Res 171, 251–260. 16707173 10.1016/j.bbr.2006.03.043

[47] Zhang Q , Li X , Cui X and Zuo P (2005) D‐galactose injured neurogenesis in the hippocampus of adult mice. Neurol Res 27, 552–556. 15978184 10.1179/016164105X25126

[48] Zhong Y , Hu Y , Peng W , Sun Y , Yang Y , Zhao X , Huang X , Zhang H and Kong W (2012) Age‐related decline of the cytochrome c oxidase subunit expression in the auditory cortex of the mimetic aging rat model associated with the common deletion. Hear Res 294, 40–48. 23022596 10.1016/j.heares.2012.09.006

[49] Salcedo‐Tello P , Hernandez‐Ortega K and Arias C (2014) Susceptibility to GSK3beta‐induced tau phosphorylation differs between the young and aged hippocampus after Wnt signaling inhibition. J Alzheimer's Dis 39, 775–785. 24270208 10.3233/JAD-130749

[50] Forde JE and Dale TC (2007) Glycogen synthase kinase 3: a key regulator of cellular fate. Cell Mol Life Sci 64, 1930–1944. 17530463 10.1007/s00018-007-7045-7 PMC11136264

[51] Tejeda‐Munoz N and Robles‐Flores M (2015) Glycogen synthase kinase 3 in Wnt signaling pathway and cancer. IUBMB Life 67, 914–922. 26600003 10.1002/iub.1454

[52] He P and Shen Y (2009) Interruption of beta‐catenin signaling reduces neurogenesis in Alzheimer's disease. J Neurosci 29, 6545–6557. 19458225 10.1523/JNEUROSCI.0421-09.2009 PMC3618977

[53] Orellana AM , Vasconcelos AR , Leite JA , de Sá Lima L , Andreotti DZ , Munhoz CD , Kawamoto EM and Scavone C (2015) Age‐related neuroinflammation and changes in AKT‐GSK‐3β and WNT/β‐CATENIN signaling in rat hippocampus. Aging (Albany NY) 7, 1094–1111. 26647069 10.18632/aging.100853 PMC4712335

[54] Kim H , Won S , Hwang DY , Lee JS , Kim M , Kim R , Kim W , Cha B , Kim T , Kim D *et al* (2011) Downregulation of Wnt/beta‐catenin signaling causes degeneration of hippocampal neurons in vivo. Neurobiol Aging 32 (2316), e1–e15. 10.1016/j.neurobiolaging.2010.03.013 20409609

[55] Cuatrecasas P and Segal S (1966) Galactose conversion to D‐xylulose: an alternate route of galactose metabolism. Science 153, 549–551. 5938779 10.1126/science.153.3735.549

[56] Lamarre M and Desrosiers RR (2008) Up‐regulation of protein L‐isoaspartyl methyltransferase expression by lithium is mediated by glycogen synthase kinase‐3 inactivation and beta‐catenin stabilization. Neuropharmacology 55, 669–676. 18582478 10.1016/j.neuropharm.2008.05.033

[57] Heo JS , Lee SY and Lee JC (2010) Wnt/beta‐catenin signaling enhances osteoblastogenic differentiation from human periodontal ligament fibroblasts. Mol Cells 30, 449–454. 20848229 10.1007/s10059-010-0139-3

[58] Lauing KL , Sundaramurthy S , Nauer RK and Callaci JJ (2014) Exogenous activation of Wnt/beta‐catenin signaling attenuates binge alcohol‐induced deficient bone fracture healing. Alcohol Alcohol 49, 399–408. 24627571 10.1093/alcalc/agu006 PMC4060733

[59] Chan DW , Mak CS , Leung TH , Chan KK and Ngan HY (2012) Down‐regulation of Sox7 is associated with aberrant activation of Wnt/b‐catenin signaling in endometrial cancer. Oncotarget 3, 1546–1556. 23295859 10.18632/oncotarget.667 PMC3681493

[60] Kaga S , Zhan L , Altaf E and Maulik N (2006) Glycogen synthase kinase‐3beta/beta‐catenin promotes angiogenic and anti‐apoptotic signaling through the induction of VEGF, Bcl‐2 and survivin expression in rat ischemic preconditioned myocardium. J Mol Cell Cardiol 40, 138–147. 16288908 10.1016/j.yjmcc.2005.09.009

[61] Nguyen P , Lee S , Lorang‐Leins D , Trepel J and Smart DK (2014) SIRT2 interacts with beta‐catenin to inhibit Wnt signaling output in response to radiation‐induced stress. Mol Cancer Res 12, 1244–1253. 24866770 10.1158/1541-7786.MCR-14-0223-T PMC4163538

[62] Gao FL , Li WS , Liu CL and Zhao GQ (2013) Silencing Bmi‐1 enhances the senescence and decreases the metastasis of human gastric cancer cells. World J Gastroenterol 19, 8764–8769. 24379598 10.3748/wjg.v19.i46.8764 PMC3870526

[63] Kim RH , Lieberman MB , Lee R , Shin KH , Mehrazarin S , Oh JE , Park NH and Kang MK (2010) Bmi‐1 extends the life span of normal human oral keratinocytes by inhibiting the TGF‐beta signaling. Exp Cell Res 316, 2600–2608. 20630502 10.1016/j.yexcr.2010.04.013 PMC2924923

[64] De Jonge HJM , Woolthuis CM , de Bont ESJM and Huls G (2009) Paradoxical down‐regulation of p16(INK4a) mRNA with advancing age in acute myeloid leukemia. Aging (Albany NY) 1, 949–953. 20157576 10.18632/aging.100096 PMC2815746

[65] Mudhasani R , Zhu Z , Hutvagner G , Eischen CM , Lyle S , Hall LL , Lawrence JB , Imbalzano AN and Jones SN (2008) Loss of miRNA biogenesis induces p19Arf‐p53 signaling and senescence in primary cells. J Cell Biol 181, 1055–1063. 18591425 10.1083/jcb.200802105 PMC2442212

[66] Xu Y , Wang Q , Li D , Wu Z , Li D , Lu K , Zhao Y and Sun Y (2016) Protective effect of lithium chloride against hypoglycemia‐induced apoptosis in neuronal PC12 cell. Neuroscience 330, 100–108. 27241942 10.1016/j.neuroscience.2016.05.047

[67] Bush AL and Hyson RL (2006) Lithium increases bcl‐2 expression in chick cochlear nucleus and protects against deafferentation‐induced cell death. Neuroscience 138, 1341–1349. 16413133 10.1016/j.neuroscience.2005.11.031 PMC1847354

[68] Liechti FD , Stüdle N , Theurillat R , Grandgirard D , Thormann W and Leib SL (2014) The mood‐stabilizer lithium prevents hippocampal apoptosis and improves spatial memory in experimental meningitis. PLoS One 9, e113607. 25409333 10.1371/journal.pone.0113607 PMC4237452

[69] Zhao XY , Sun JL , Hu YJ , Yang Y , Zhang WJ , Hu Y , Li J , Sun Y , Zhong Y , Peng W *et al* (2013) The effect of overexpression of PGC‐1alpha on the mtDNA4834 common deletion in a rat cochlear marginal cell senescence model. Hear Res 296, 13–24. 23159434 10.1016/j.heares.2012.11.007

